# PIM-Related Kinases Selectively Regulate Olfactory Sensations in *Caenorhabditis elegans*

**DOI:** 10.1523/ENEURO.0003-19.2019

**Published:** 2019-08-22

**Authors:** Karunambigai S. Kalichamy, Kaisa Ikkala, Jonna Pörsti, Niina M. Santio, Joel Tuomaala, Sweta Jha, Carina I. Holmberg, Päivi J. Koskinen

**Affiliations:** 1 Section of Physiology and Genetics, Department of Biology, University of Turku, 20500 Turku, Finland; 2 Research Programs Unit, Translational Cancer Biology Program, University of Helsinki, 00290 Helsinki, Finland

**Keywords:** chemosensory neurons, chemotaxis, gustation, PIM kinases, olfaction, phosphorylation

## Abstract

The mammalian PIM family of serine/threonine kinases regulate several cellular functions, such as cell survival and motility. Because PIM expression is observed in sensory organs, such as olfactory epithelium, we now wanted to explore the physiological roles of PIM kinases there. As our model organism, we used the *Caenorhabditis elegans* nematodes, which express two PIM-related kinases, PRK-1 and PRK-2. We demonstrated PRKs to be true PIM orthologs with similar substrate specificity as well as sensitivity to PIM-inhibitory compounds. When we analyzed the effects of pan-PIM inhibitors on *C. elegans* sensory functions, we observed that PRK activity is selectively required to support olfactory sensations to volatile repellents and attractants sensed by AWB and AWC^ON^ neurons, respectively, but is dispensable for gustatory sensations. Analyses of *prk*-deficient mutant strains confirmed these findings and suggested that PRK-1, but not PRK-2 is responsible for the observed effects on olfaction. This regulatory role of PRK-1 is further supported by its observed expression in the head and tail neurons, including AWB and AWC neurons. Based on the evolutionary conservation of PIM-related kinases, our data may have implications in regulation of also mammalian olfaction.

## Visual Abstract

**Figure:**
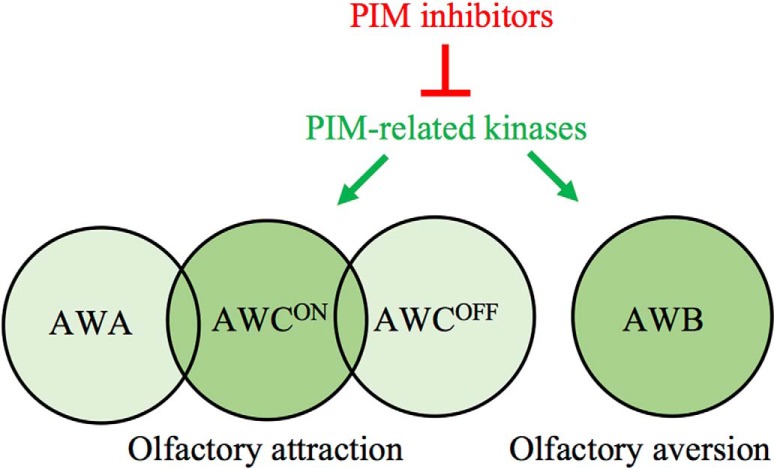

## Significance Statement

The *Caenorhabditis elegans* nematodes provide a useful model organism to study evolutionarily conserved physiological phenomena, such as the roles of kinases in modulation of chemosensory functions, because >80% of mammalian kinases have orthologs there, including also PIM kinases. This study reveals that the invertebrate PIM-related kinases can regulate olfaction, prompting similar studies also with mammalian PIM kinases.

## Introduction

The mammalian PIM family of serine/threonine kinases consists of three highly homologous members, PIM-1, PIM-2, and PIM-3 (Brault et al., 2010; Nawijn et al., 2011; Santio and Koskinen, 2017). As the acronym indicates, the *PIM* family genes were originally identified as proviral integration sites for the Moloney murine leukemia virus. The abundance of the constitutively active PIM kinases is normally tightly regulated, mainly through the JAK/STAT pathway of Janus kinases and signal transducers and activators of transcription. However, abnormally elevated PIM expression and activity levels are observed in hematological malignancies and solid tumors, where PIM kinases support cancer cell survival, motility, and metastatic growth. These effects are mediated by phosphorylation of multiple substrates, such as the transcriptional regulators NFATc1 (nuclear factor of activated T cells; Rainio et al., 2002; Santio et al., 2010) and NOTCH1 (Santio et al., 2016), and the pro-apoptotic BAD protein (Yan et al., 2003; Aho et al., 2004). PIM kinase activity can be antagonized by inhibitory compounds (Brault et al., 2010; Santio and Koskinen, 2017), which may have therapeutic potential, but also provide research tools.

The functions of PIM kinases in normal tissues have been studied less extensively. In addition to hematopoietic and epithelial cells, *pim* mRNA expression has been observed in neuronal tissues of developing mouse and quail embryos, including sensory organs such as neural retina and olfactory epithelium (Eichmann et al., 2000). This has raised the questions of whether PIM kinases are required for the development of these organs and/or whether they regulate sensory cell functions. Therefore, we have now conducted behavioral studies in the nematode *Caenorhabditis elegans*, which was chosen as our model organism for several reasons. First of all, the fully sequenced and annotated *C. elegans* genome has family homologs for >80% of the human kinome, including also PIM kinases (Manning, 2005). Furthermore, its nervous system provides a well-defined framework for studies on sensory functions. In the head of *C. elegans*, there are chemosensory neurons that enable it to discriminate between beneficial and harmful substances in its living environment, and thereby help it to find food and mating partners as well as to avoid danger (Bargmann et al., 1993). The sensed environmental cues cause changes in the overall motility of the animals, eliciting chemotactic movements toward or away of them.

Volatile compounds can be sensed via three pairs of olfactory neurons (AWA, AWB, and AWC), whereas water-soluble compounds are sensed by gustatory neurons (ASE and ASH), which are responsive to both chemical and mechanical cues (Bargmann, 2006). AWA neurons detect attractive odorants like 2,4,5-trimethylthiazole (TMT), diacetyl and pyrazine, whereas AWC neurons sense butanone, isoamyl alcohol (IAA), benzaldehyde, 2,3-pentanedione, and TMT (Bargmann et al., 1993). ASE neurons detect soluble attractants like sodium or potassium chloride (Pierce-Shimomura et al., 2001), whereas AWB neurons react to volatile repellents like 2-nonanone or 1-octanol (Troemel et al., 1997), and ASH to soluble repellents like copper solutions (Bargmann, 2006). The bilaterally symmetrical pairs of chemosensory neurons are structurally similar on each side of the animals. However, AWC and ASE amphid neurons are functionally distinct between the right and left sides and detect different compounds (Pierce-Shimomura et al., 2001; Wes and Bargmann, 2001).

In the *C. elegans* chemosensory neurons, the signals are received by G-protein-coupled receptors that use cGMP or polyunsaturated fatty acids as second messengers to open cation channels and depolarize the neurons (Bargmann, 2006). These pathways may be modulated by intracellular enzymes, such as PIM-related kinases (PRKs). *C. elegans* has two homologs for the three mammalian PIM kinases, PRK-1 and PRK-2 (wormbase.org). Very little is known about the expression or functions of PRKs, except that PRK-1 is expressed in the intestine as well as head and tail neurons (wormbase.org), and that PRK-2 regulates neurite branching (Zheng et al., 2011).

In this study, we confirmed that PRKs are functional orthologs of mammalian PIM kinases. By using PIM-selective inhibitors as well as *prk* mutant strains, we obtained evidence that PRKs do not affect gustatory sensations, but selectively target AWC^ON^ and AWB neurons to regulate olfactory sensations. Furthermore, we show that PRK-1 is expressed in these neurons.

## Materials and Methods

### *C. elegans* culture and strains

The *C. elegans* strains were grown and maintained on NGM agar plates seeded with *E.coli* (RRID:WB-STRAIN:OP50-1), using standard culturing methods (Brenner, 1974). The wild-type strain (RRID:WB-STRAIN:N2-ancestral) and the mutant strains *prk-1(pk86::Tc1)III* (RRID:WB-STRAIN:NL1100), *prk-2(ok3069)III* (RRID:WB-STRAIN:RB2267), *tax-2(p691)I* (RRID:WB-STRAIN:PR691), *oyIs44* [*odr-1::RFP* + *lin-15*(+)] (RRID:WB-STRAIN:PY2417) and *dpy-5(e907)I*; *sEx14881* [*rCesC06E8.3A::GFP* + *pCeh361*] (RRID:WB-STRAIN:BC14881) strain with GFP expression driven by the *prk-1* promoter (McKay et al., 2003) were obtained from the Caenorhabditis Genetics Center. The mutated *prk-1* gene in the *prk-1(pk86)* strain was amplified with the primers 5′GTCGGATCCATGATCAAACGAA3′ and 5′TGGCTCGAGTTCTGTGTCAA3′ and sequenced. The double-transgenic strain *sEx14881*; *oyIs44* (PJK001) was generated by conventional crossing and screening methods.

### DNA constructs

Glutathione S-transferase (GST) fusion constructs were prepared to be able to produce PRK proteins in bacteria. For this purpose, a cDNA library was prepared from wild-type *C. elegans* samples using the Maxima H minus first strand synthesis kit (Thermo Fisher Scientific) according to the manufacturer’s instructions. The *prk-1* cDNA (isoform A, amino acids 1–530) was amplified from there with the *prk-1* primers described in the previous section, and ligated into the pGEX-6P-1 plasmid (GE Healthcare) between BamHI and XhoI sites. Full-length *prk-2* cDNA was subcloned from the *rab-3:prk-2* plasmid (Zheng et al., 2011; kindly provided by Michael Nonet, Washington University, St. Louis, MO), and ligated between EcoRI and SmaI sites in the pGEX-6P-2 plasmid. Preparation of GST-tagged human PIM1 (full-length short isoform) has been previously described (Santio et al., 2016), and GST-tagged human NFATC1 (amino acids 1–418) was a kind gift from S. N. Ho (Stanford University, Stanford, CA).

### Phosphorylation assays

GST fusion proteins were produced in bacteria, purified with glutathione sepharose beads (GE Healthcare) and either left uncleaved or cleaved with PreScission protease (GE Healthcare) according to the manufacturer’s instructions. To inhibit kinase activity, PIM or PRK proteins were pre-incubated for 10–20 min with PIM-selective inhibitors, which had been dissolved in dimethyl sulfoxide (DMSO) at 10 µm concentration. Three structurally unrelated PIM-selective inhibitors were used: the pyrrolocarbazole carbaldehyde DHPCC-9 (Akué-Gédu et al., 2009; kindly provided by Pascale Moreau, Université Clermont Auvergne, France), the imidazopyridazine SGI-1776 (SelleckChem) or the thiazolidinedione AZD-1208 (MedChemExpress). 0.1% DMSO alone was used as a negative control.

Radioactive *in vitro* kinase assays were conducted in the presence of γ-^32^P-ATP (GE Healthcare) as previously described (Kiriazis et al., 2013). Phosphorylated proteins were resolved in 10% SDS-PAGE and stained with the PageBlue solution (Thermo Fisher Scientific) to visualize protein loading. Radioactivity of the samples was analyzed by autoradiography. Non-radioactive kinase assays were performed similarly, but without γ-^32^P-ATP. After separation on 10% SDS-PAGE, proteins were blotted onto PVDF membrane (EMD Millipore). Total proteins were stained by the REVERT Total Protein Stain Kit (926-11010; LI-COR Biosciences), after which phosphorylated proteins were detected with the phospho-RXXS*/T* antibody (RRID:AB_331810#9614; Cell Signaling Technology). From the PRK-1 amino acid sequence, four putative target sites for the antibody were found: 72–75 (RSWT), 382–385 (RSES), 451–454 (RKKS), and 515–518 (RKGS). The signal intensities were quantitated by ImageJ software (RRID:SCR_003070; National Institutes of Health). Signals from phosphorylated samples were compared with the amounts of total protein to calculate relative phosphorylation levels.

### Behavioral assays

Well-fed Day 1 *C. elegans* adults synchronized at 20°C were exposed in S-basal buffer to indicated concentrations of the PIM inhibitors (DHPCC-9, SGI-1776, or AZD-1208) dissolved in DMSO; 0.1% DMSO alone was used as a negative control. After 120 min of exposure, animals were washed twice with the buffer and once with distilled water, and subjected to olfactory or gustatory assays.

#### Olfactory assays

The protocol for olfactory assays was adopted from Bargmann et al. (1993). The volatile odorants were diluted in ethanol, which was used as the neutral control. Chemotaxis assays were conducted with indicated concentrations of butanone, benzaldehyde, IAA, 2,3-pentanedione, TMT, diacetyl, pyrazine or 1-octanol (Sigma-Aldrich). One microliter of 1 m sodium azide (Sigma-Aldrich) was dropped in advance to both odorant and control spots to paralyze animals reaching them. Assay plates with 150–200 animals were incubated for 120 min at 20°C, after which plates were moved to 4°C to stop animal movements. Chemotaxis indices (CI = O − C/O + C) were calculated as the number of animals that moved toward the attractive odorant (O) minus the number of animals toward the control (C), divided by the total number of animals. Avoidance indices were calculated similarly, but taken into account the number of animals that moved away from the repellent.

#### Gustatory assays

The protocol for gustatory assays with water-soluble attractants was adopted from Jansen et al. (2002) with slight modifications. Four quadrant lines were drawn on agar plates. Twenty-five microliter aliquots of 2.5 m NaCl or KCl (Sigma-Aldrich) dissolved in distilled water were dropped on the opposite quadrants (A, C) and the solvent as control on the other pair of quadrants (B, D), after which the plates were allowed to dry at room temperature for 60 min. Control- and drug-exposed animals were washed and dropped in the middle of the assay plate and incubated for 60 min at 20°C. Then 0.5 µl of 1 m sodium azide was added to the salt or solvent spots to paralyze the animals. Animals at each quadrant were counted and calculated for chemotaxis index (CI = (A + C) – (B + D)/(A + B + C + D) as number of animals on the salt (A + C) minus number of animals on the solvent (B + D) divided by the total number of animals.

Aversion assays were performed according to Wicks et al. (2000) against water-soluble copper ions. Twenty-five microliter aliquots of 50 and 100 mM CuSO_4_ (Sigma-Aldrich) dissolved in water were pipetted across the midlines of the assay plates. Approximately 150–200 animals were placed on one side and odorants or control spots on the other side. After 120 min of incubation at 20°C, chemotactic indices (CI = A/A + B) were calculated as the number of animals that had crossed the aversion compound line (A) divided by the total number of animals.

### Microscopy

For imaging, animals were mounted on 2% agarose pads on glass slides and paralyzed with 0.2 mM levamisole hydrochloride solution (Sigma-Aldrich). Young adult hermaphrodites were imaged by the Cytation 5 multi-mode reader (BioTek) with automated digital wide-field microscopy with 10× or 20× objectives.

### Statistical analysis

All analyses of behavioral data were performed with IBM SPSS statistics 24 software (RRID:SCR_002865), using one-way ANOVA and four multiple-comparison *post hoc* tests (Bonferroni test, Fischer’s least significant difference test, Tukeýs honestly significant difference test, and Dunnett’s T3 test). Asterisks in the graphs or box plots indicate that the mean differences were statistically significant (*p* < 0.01) in all four *post hoc* tests.

## Results

### Mammalian PIM Kinases and *C. elegans* PRKs are true orthologs

**Figure 1.**
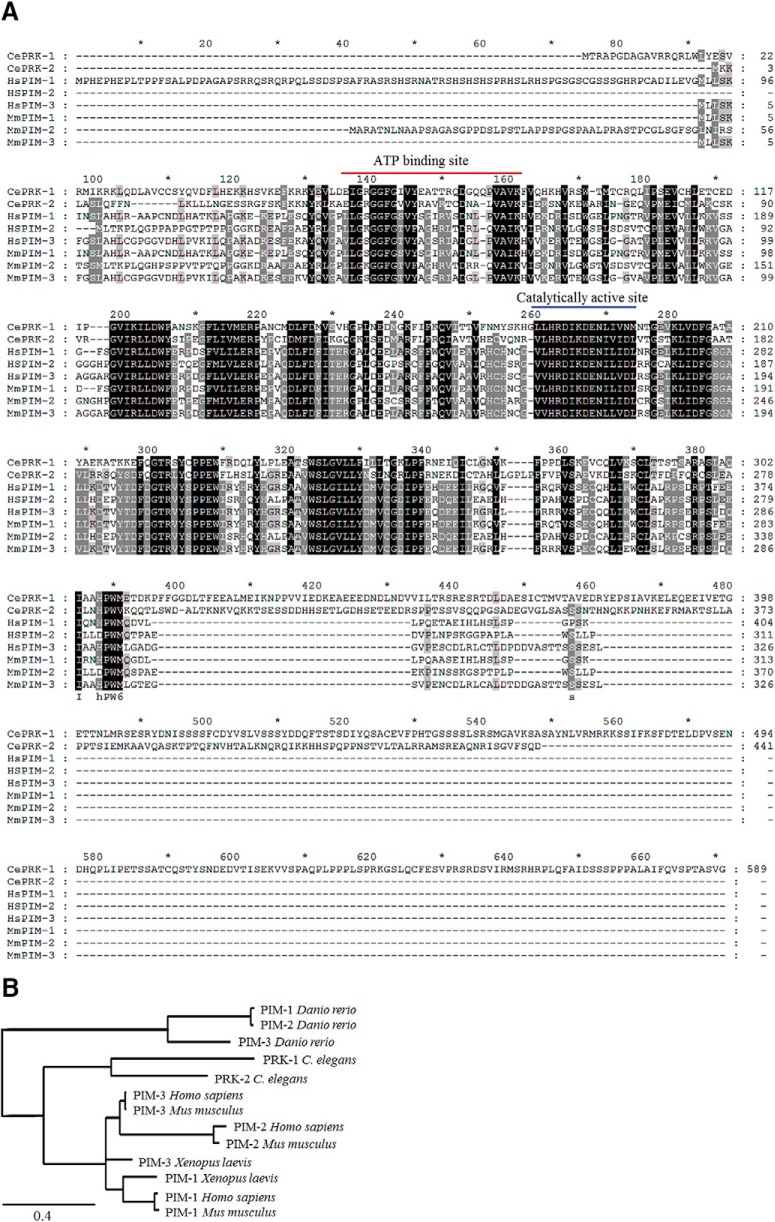
Mammalian PIM kinases and *C. elegans* PRKs are true orthologs. ***A***, Amino acid sequences of *C. elegans* PRK-1 (NM_001276777.1) and PRK-2 (CAA84323.2) were aligned with *Homo sapiens* PIM-1 (NP_001230115.1), PIM-2 (NP_006866.2), and PIM-3 (NP_001001852.2), and *Mus musculus* PIM-1 (NP_032868.2), PIM-2 (NP_613072.1), and PIM-3 (NP_663453.1), using the Clustal X 2.0 software (Thompson et al., 1997). Identical or similar amino acids present in all or only in some of them are marked with black or gray backgrounds, respectively. Positions of the conserved ATP-binding and catalytically active sites are highlighted with red and blue lines, respectively. ***B***, The amino acid sequences of the *C. elegans* (PRK-1 and PRK-2), *Danio rerio* (PIM-1, PIM-2, and PIM-3), *Xenopus laevis* (PIM-1 and PIM-3), *Mus musculus* (PIM-1, PIM-2, and PIM-3) and *Homo sapiens* (PIM-1, PIM-2, and PIM-3) were used to draw a phylogenetic tree of PIM orthologs and paralogs in distinct species (http://www.phylogeny.fr). The scale of 0.4 refers to 40% difference between sequences.

The *C. elegans prk-1* and *prk-2* genes that encode PRKs had previously been mapped to chromosome III and sequenced (Ronald Plasterk, The Netherlands; wormbase.org). An amino acid sequence alignment between the two *C. elegans* PRKs and the three human or mouse PIM kinases revealed that they are well conserved with ∼40% amino acid similarity (Fig. 1*A*). Expectedly, the highest identities were observed within their kinase domains, especially at the ATP-binding site and the catalytically active site. By contrast, the C-terminal tails of *C. elegans* PRKs are much longer than those of their mammalian orthologs, and are not conserved even between PRK-1 and PRK-2.

A phylogenetic tree (Fig. 1*B*) shows the evolutionary distance between PIM orthologs and paralogs in several species, including also the zebrafish *Danio rerio* and the African clawed frog *Xenopus laevis*. In mammalian species and in frogs, there is more homology between each PIM ortholog than between the distinct family members, PIM-1, PIM-2, and PIM-3. By contrast, the *C. elegans* and *D. rerio* orthologs are all significantly separated from the other species, with *C. elegans* proteins however being more homologous to mammalian ones than those from *D. rerio*. Interestingly and maybe also surprisingly, no obvious PIM orthologs have been found in the fruit fly *Drosophila melanogaster* (Manning, 2005; Peter Gallant, University of Würzburg, Germany, personal communication).

**Figure 2.**
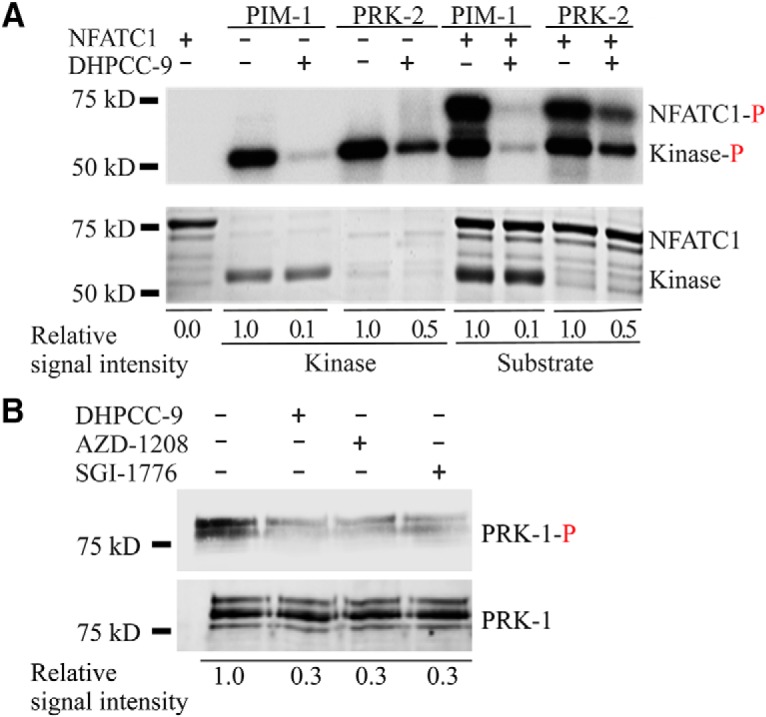
PIM and PRK kinases function similarly *in vitro.* ***A***, Radioactive kinase assays were performed to analyze the *in vitro* ability of human PIM-1 and *C. elegans* PRK-2 to phosphorylate themselves or the PIM substrate NFATC1 (amino acids 1–418) in the absence (−) or presence (+) of 10 μm DHPCC-9. The intensities of phosphorylated proteins are shown at the top and the total amounts of proteins at the bottom. Shown are also the relative levels of phosphorylation of kinases and their substrates in control versus drug-treated samples in this dataset representing three independent experiments. ***B***, PRK-1 autophosphorylation was analyzed in the absence (−) or presence (+) of 10 μm DHPCC-9, AZD-1208, or SGI-1776. The phosphorylated protein was visualized by immunoblotting with the phospho-RXXS*/T* antibody, which has several potential target sites in PRK-1.

To demonstrate that the *C. elegans* PRKs are functional orthologs for mammalian PIM kinases, we took human PIM-1 and *Ce* PRK-2 proteins as representatives of each family and analyzed for their activities by radioactive *in vitro* kinase assays. As expected and shown in Figure 2*A*, both PIM-1 and PRK-2 were able to autophosphorylate themselves. More interestingly, also PRK-2 was able to phosphorylate known PIM substrates, such as NFATc1, suggesting that PIMs and PRKs are true orthologs sharing similar preferences for their target sites.

Part of the phosphorylation samples had been pretreated with the pyrrolocarbazole carbaldehyde compound DHPCC-9, which has been shown to selectively inhibit catalytic activities of all three mammalian PIM kinases *in vitro* (Akué-Gédu et al., 2009), in cell-based assays (Santio et al., 2010) and under *in vivo* conditions in mice (Santio et al., 2015) or chicken eggs (Santio et al., 2016). At the 10 µm concentration used, DHPCC-9 was able to inhibit autophosphorylation of not only PIM-1, but also PRK-2 (Fig. 2*A*), indicating that the ATP-binding pockets of PIMs and PRKs are conserved enough to allow binding of the ATP-competitive drug. In addition, the abilities of both PIM-1 and PRK-2 to phosphorylate NFATC1 were compromised by DHPCC-9.

To determine whether the kinase activity of also PRK-1 can be reduced by DHPCC-9 and whether two other structurally unrelated PIM inhibitors, the imidazopyridazine SGI-1776 (Mumenthaler et al., 2009) and the thiazolidinedione AZD-1208 (Keeton et al., 2014) have similar effects in *C. elegans*, additional non-radioactive *in vitro* kinase assays were performed. There we analyzed the autophosphorylation activity of PRK-1 by immunoblotting with the phospho-RXXS*/T*-specific antibody, for which four potential target sites were identified from the PRK-1 amino acid sequence. Although PRK-1 was prominently phosphorylated in the presence of DMSO, its autophosphorylation was significantly reduced by 10 µm pretreatment with either DHPCC-9, AZD-1208 or SGI-1776 (Fig. 2*C*). Thus, although all PIM inhibitors reduced activities of PRKs less efficiently than that of PIM-1, their effects were strong enough to prompt us to use them as tools to investigate the physiological functions of PRKs in *C. elegans*.

### PIM inhibitors specifically suppress attractive olfactory responses via AWC^ON^ neurons

**Figure 3.**
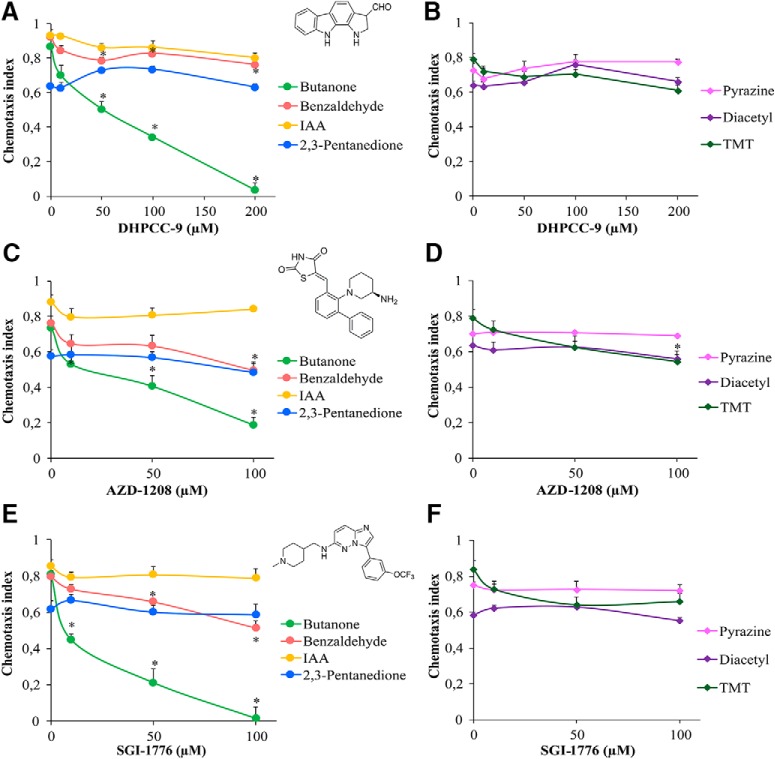
PIM inhibitors specifically suppress olfactory sensing via AWC^ON^ neurons. Synchronized young adult animals were exposed to indicated concentrations of PIM inhibitors DHPCC-9 (***A***, ***B***), AZD-1208 (***C***, ***D***), or SGI-1776 (***E***, ***F***) for 120 min. Chemotaxis assays with odorants were performed for another 120 min. Chemical structures of the PIM inhibitors are shown in between the dose-dependent chemotactic indices of animals in response to AWC odorants (***A***, ***C***, ***E***) butanone (1:1000), benzaldehyde (1:200), IAA (1:100), and 2,3-pentanedione (1:10000), or AWA odorants (***B***, ***D***, ***F***) diacetyl (1:1000), pyrazine (10 mg/ml), and TMT (1:1000). Each data point represents the mean of at least six independent experiments with 150–200 animals per assay. Error bars indicate SEM and asterisks statistically significant differences (*p* < 0.01) between control and drug-exposed animals.

We previously reported that mammalian PIM kinases are expressed in sensory organs, such as the olfactory epithelium (Eichmann et al., 2000). Because PRK-1, and possibly also PRK-2, are expressed in *C. elegans* head neurons, we wanted to investigate whether PRKs are involved in regulation of olfaction. Before carrying out behavioral assays, we experimentally determined that the maximal tolerated dosage of the PIM inhibitor DHPCC-9 in *C. elegans* is ∼ 200 µm. Then we synchronized wild-type animals, exposed populations of Day 1 adults to 10-200 µm concentrations of DHPCC-9 for 120 min, and conducted chemotaxis assays to AWC-sensed attractive odorants for another 120 min. At tested dilutions of odorants, control animals vigorously responded to butanone, benzaldehyde and isoamyl alcohol (IAA) with chemotaxis indices (CIs) of ∼ 0.9, whereas 2,3-pentanedione was less attractive with CI of 0.6 (Fig. 3*A*). More interestingly, animals pre-exposed to increasing concentrations of DHPCC-9 consistently displayed a significant dose-dependent decrease in their chemotaxis to butanone. The inhibitory effect of DHPCC-9 on the chemotactic behavior became evident already at the lowest 10 µm concentration. However, it was most prominent at the maximal dose of 200 µm, where animals were moving randomly to all directions, as if they did not sense the presence of the odorant. There was a statistically significant reduction (∼20%) in chemotaxis to benzaldehyde in DHPCC-9-treated animals compared with controls, whereas hardly any effects were observed by DHPCC-9 on chemotaxis to IAA or 2,3-pentanedione. It is known that butanone is sensed by AWC^ON^ neurons, 2,3-pentanedione by AWC^OFF^ neurons and benzaldehyde and IAA by both (Troemel et al., 1999). Thus, our data suggest that inhibition of PRK activity by DHPCC-9 specifically and asymmetrically suppresses olfactory sensing via AWC^ON^ neurons.

To examine whether DHPCC-9 affects the olfactory functions of also AWA neurons, we performed chemotaxis assays with AWA-sensed attractive odorants pyrazine, diacetyl and 2,4,5-trimethylthiazole (TMT). No significant suppression was observed for pyrazine or diacetyl by DHPCC-9, but the response to TMT, which can be sensed also by AWC neurons (Bargmann et al., 1993), was slightly reduced (∼15%) compared with controls (Fig. 3*B*).

To confirm that the observed effects of DHPCC-9 were dependent on its ability to inhibit endogenous PRK activity and were not just because of some unspecific off-target effects, we repeated the chemotaxis analyses by using the two other tested PIM inhibitors, SGI-1776 and AZD-1208. AZD-1208 efficiently targets all three PIM family members (Keeton et al., 2014) and is more selective than SGI-1776, which in addition to PIM-1 and PIM-3 also inhibits FLT-3 and haspin (Mumenthaler et al., 2009). These two inhibitors appeared to be more toxic than DHPCC-9, so animals were exposed to maximally 100 µm concentrations of them. When animals pre-exposed to either AZD-1208 or SGI-1776 were assayed for their chemotactic responses to attractive odorants sensed by either AWA or AWC neurons, very similar dose-dependent data were obtained as with DHPCC-9 (Fig. 3*C*–*F*). Again, chemotaxis to the AWC^ON^-sensed butanone was significantly suppressed by both AZD-1208 and SGI-1776. Chemotaxis to benzaldehyde and TMT was also reduced (∼20%) compared with controls, whereas no significant effects were observed in animals treated with AWC^OFF^-sensed 2,3-pentanedione or AWA-sensed pyrazine and diacetyl. Overall, our data suggest that the observed suppressive effects of all the drugs on olfactory sensations via AWC^ON^ neurons are because of their shared ability to inhibit activities of the PRKs.

### Inhibition of butanone sensing is reversible and dependent on odorant dosage

**Figure 4.**
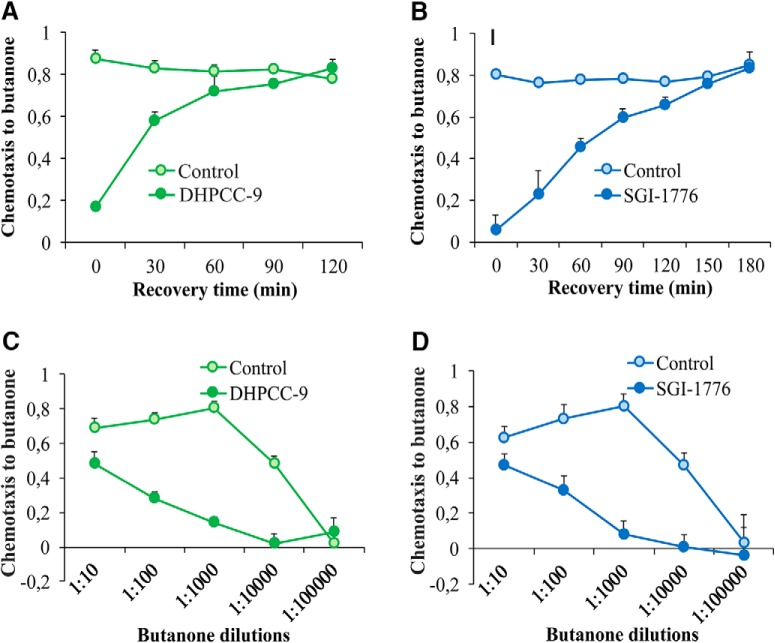
Inhibition of butanone sensing is reversible and dependent on odorant dosage. Animals exposed for 120 min to 200 μm DHPCC-9 (***A***, ***C***) or 100 μm SGI-1776 (***B***, ***D*)** were assayed for chemotaxis to butanone (1:1000) after indicated intervals of recovery times (***A***, ***B***), or to indicated dilutions of butanone (***C***, ***D***). Each data point represents the means and SEM of four independent experiments with 150–200 animals per assay.

To determine whether PRK inhibition resulted in permanent or only temporary changes in neuronal activity, animals were exposed for 120 min to 200 µm DHPCC-9 or 100 µm SGI-1776, and then allowed to recover for up to 180 min before assaying their chemotaxis to butanone. As shown in Figure 4, *A* and *B*, drug-exposed animals quickly regained their ability to sense butanone, with complete recovery within 120 or 180 min after exposure to DHPCC-9 or SGI-1776, respectively. These results indicate that the suppressive effects of the PIM inhibitors on olfactory sensations are reversible. Furthermore, the rapid inhibitory responses as well as recovery times suggest that the PIM inhibitors have direct, although as yet uncharacterized effects on olfactory signaling in the AWC^ON^ neurons.

To investigate the effects of the PIM inhibitors on butanone sensing in more detail, drug-exposed animals and their controls were assayed for their chemotaxis to a wide range of butanone dilutions. As shown in Figure 4, *C* and *D*, high concentrations of butanone were able to overcome the inhibitory effects of both DHPCC-9 and SGI-1776, whereas at lower concentrations, the chemotactic behavior of the animals was lost even without inhibition. Thus, these results confirmed that the 1:1000 dilution used in our other assays had been optimal to reveal the suppressive effects of the PIM inhibitors on butanone sensing.

### PRK-1 is more important than PRK-2 for olfactory responses via AWC neurons

**Figure 5.**
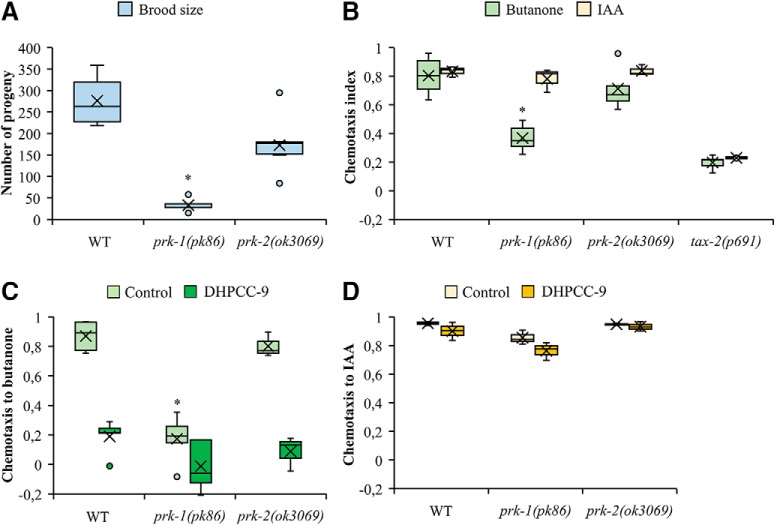
PRK-1 expression and activity is required for butanone sensing. ***A***, Reproductive capacities of wild-type (WT; *n* = 13), *prk-1(pk86)* (*n* = 5), and *prk-2(ok3069)* (*n* = 9) animals, as determined by the amounts of eggs laid by each parent. Shown in the box plots are mean (cross), median, quartiles (boxes), and range (whiskers). The asterisks indicate statistically significant differences (*p* < 0.01) between wild-type and mutant animals. ***B***, Wild-type and mutant animals were tested for chemotaxis toward butanone (1:1000) or IAA (1:100). *tax-2* was used as a chemotaxis-deficient control strain. For butanone and IAA, the box plot data are derived from five and three independent experiments, respectively, with 100–150 animals per assay. The asterisks indicate statistically significant differences (*p* < 0.01) between wild-type and mutant animals. ***C***, ***D***, The same strains were also exposed for 120 min to 200 μm DHPCC-9 and then tested as above toward butanone (***C***) or IAA (***D***). The asterisks indicate statistically significant differences (*p* < 0.01) between wild-type and mutant animals.

To evaluate the contribution of individual PRK family members to the olfactory responses, we acquired mutant strains for both of them. The *prk-1(pk86)* strain contains a Tc1 transposon inserted in the coding region of the *prk-1* gene. Based on our sequencing data, this insertion had resulted in a complex rearrangement of nucleotides from exon 2 to exon 3, replacing Lys123 with a stop codon. Thus, the mutated gene is expected to produce a truncated, catalytically inactive protein. In the *prk-2(ok3069*) strain in turn, there is a 617 bp deletion from the first intron to the third exon of the *prk-2* gene (Zheng et al., 2011), which is expected to destroy the catalytic activity of the protein. The *prk-1(pk86) and prk-2(ok3069)* mutant animals are viable, but we noticed that their populations propagate abnormally slow. To analyze the effects of the mutations on the reproductive capacity in more detail, brood size assays were performed. When we measured the mean amounts of eggs laid by synchronized gravid adult individuals, the average brood size was reduced in both mutant strains, but more remarkably in the *prk-1(pk86)* strain (Fig. 5*A*).

We then analyzed the chemotaxis of the *prk* mutants toward AWC-sensed attractive odorants, butanone and IAA. There both *prk-1(pk86)* and *prk-2(ok3069)* mutant animals responded normally to IAA, but only the *prk-1(pk86)* animals were significantly defective in their responses to butanone (Fig. 5*B*). The *tax-2(p691)* strain was used there as a chemotaxis-defective negative control, because it lacks expression for the TAX-2 cyclic nucleotide-gated channel ß subunit required to detect AWC-sensed odorants (Bargmann, 2006).

In another independent set of experiments, we pretreated wild-type and *prk* mutant strains with the PIM inhibitor DHPCC-9, after which we conducted chemotaxis assays to butanone and IAA. As expected based on previous results, the *prk-1* mutation and/or DHPCC-9 blocked responses to butanone, but not to IAA (Fig. 5*C*,*D*). These data suggest that most of the effects of the PIM inhibitor on butanone sensing were mediated via inhibition of PRK-1 activity.

### PRK-1 regulates also repulsive olfactory responses via AWB neurons

**Figure 6.**
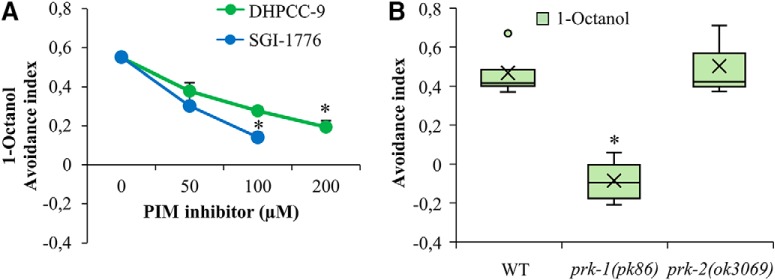
PRK-1 is essential also for sensing of olfactory repellents via AWB neurons. ***A***, Wild-type animals were treated for 120 min with indicated concentrations of DHPCC-9 or SGI-1776. Aversion assays with 1-octanol (1:100) were performed for another 120 min. Shown are dose-dependent avoidance indices. Each data point represents the mean and SEM of at least four independent experiments with 100–150 animals per assay. The asterisks indicate statistically significant differences (*p* < 0.01) between control- and drug-exposed animals. ***B***, WT, *prk-1(pk86)*, and *prk-2(ok3069)* animals were similarly tested for aversion to 1-octanol. Shown are box plot data collected from three independent experiments with 100–150 animals per assay. The asterisks indicate statistically significant differences (*p* < 0.01) between wild-type and mutant animals.

To determine whether PRKs are involved in the sensation of volatile repellents by AWB neurons, wild-type animals were exposed for 120 min to increasing concentrations of DHPCC-9 or SGI-1776, and then tested for their ability to sense 1-octanol and move away from it. After 120 min incubation, the avoidance indices were calculated. As shown in Figure 6*A*, more than half of the control-treated animals tried to avoid 1-octanol. By contrast, the avoidance responses were significantly and dose-dependently reduced in the drug-exposed populations, suggesting that the PIM inhibitors are able to diminish olfactory sensing also toward repellents via AWB neurons.

When the olfactory aversion assays were repeated with mutant strains, *prk-1(pk86)* mutants showed a random chemotaxis toward either 1-octanol or the ethanol control, whereas the *prk-2(ok3069)* mutants avoided 1-octanol similarly to the wild-type animals (Fig. 6*B*). These data suggest that PRK-1 is more essential than PRK-2 for the observed effects of pan-PIM inhibitors on both attractive and repulsive olfactory responses.

### PIM inhibitors do not affect gustatory sensations

**Figure 7.**
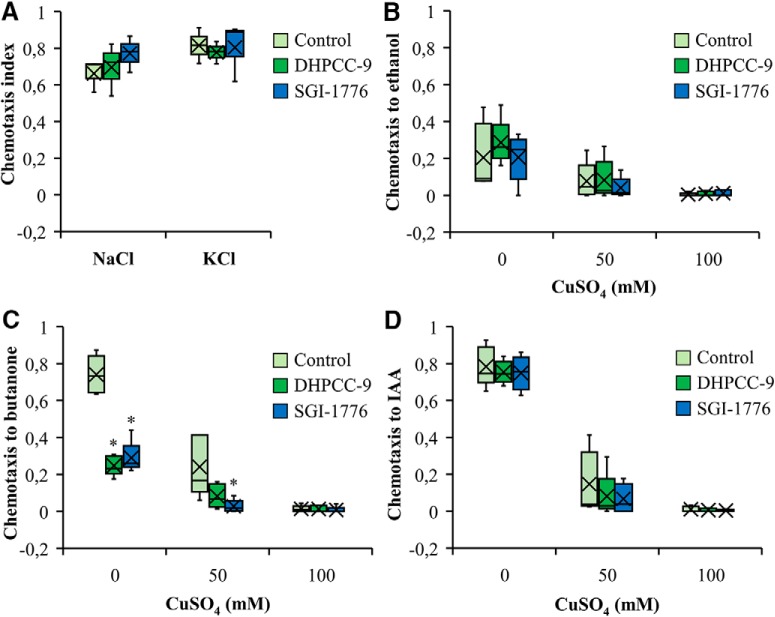
PIM inhibitors do not affect responses to gustatory attractants or repellents. Wild-type animals were treated for 120 min with 200 μm DHPCC-9 or 100 μm SGI-1776 and assayed for responses to gustatory attractants (***A***) or repellents (***B***–***D***). ***A***, Shown are box plots of chemotactic indices of control- or drug-exposed animals to 2.5 m NaCl or 2.5 m KCl. Data were collected from three independent experiments with 150–200 animals per assay. ***B***–***D***, Indicated concentrations of CuSO_4_ were pipetted across the midlines of the assay plates. Control- or drug-exposed animals were placed on one side and drops of ethanol (***B***), butanone (***C***), or IAA (***D***) to the other side. After 120 min of incubation, chemotactic indices to the odorants were calculated. Shown are box plot data collected from four independent experiments with 150–200 animals per assay. The asterisks indicate statistically significant differences (*p* < 0.01) between control- and drug-exposed animals.

We next examined whether PRKs are essential also for the attractive and aversive gustatory responses by the ASE and ASH neurons, respectively. Similarly to AWC neurons, also the ASE neurons are functionally asymmetric: the ASER (right) neuron preferentially detects potassium ions, whereas the ASEL (left) neuron detects sodium ions (Pierce-Shimomura et al., 2001). When we placed animals exposed to 200 µm DHPCC-9 or 100 µm SGI-1776 on plates containing drops of either 2.5 m NaCl or KCl, and calculated the chemotactic indices after 120 min incubation, more than half of the animals were attracted to either salt, but the PIM inhibitors did not significantly interfere with these responses compared with controls (Fig. 7*A*).

To investigate the repulsive responses by ASH neurons, midlines of distilled water or aqueous CuSO_4_ solution (50 or 100 mM) were drawn onto assay plates. Control- or drug-exposed animals were placed on one side of the midline, whereas aliquots of ethanol, butanone or IAA were dropped on the other side. After 120 min incubation, chemotactic indices were calculated. As shown in Figure 7*B*, the initially mild chemotaxis toward the neutral odorant ethanol was further reduced by increasing concentrations of CuSO_4_ in the midline, whereas the PIM inhibitors did not have significant effects. Both butanone (Fig. 7*C*) and IAA (Fig. 7*D*) efficiently attracted the animals to cross the midline of water, but the chemotactic responses were strongly diminished by the presence of 50 mM CuSO_4_ and completely blocked by 100 mM CuSO_4_. As observed also in our olfactory assays (Fig. 3*A*,*C*,*E*), exposures to either 200 µm DHPCC-9 or 100 µm SGI-1776 efficiently inhibited the positive chemotaxis toward butanone, but had less effects on IAA sensing (Fig. 7*C*,*D*).

### PRK-1 is expressed in amphid and phasmid neurons

**Figure 8.**
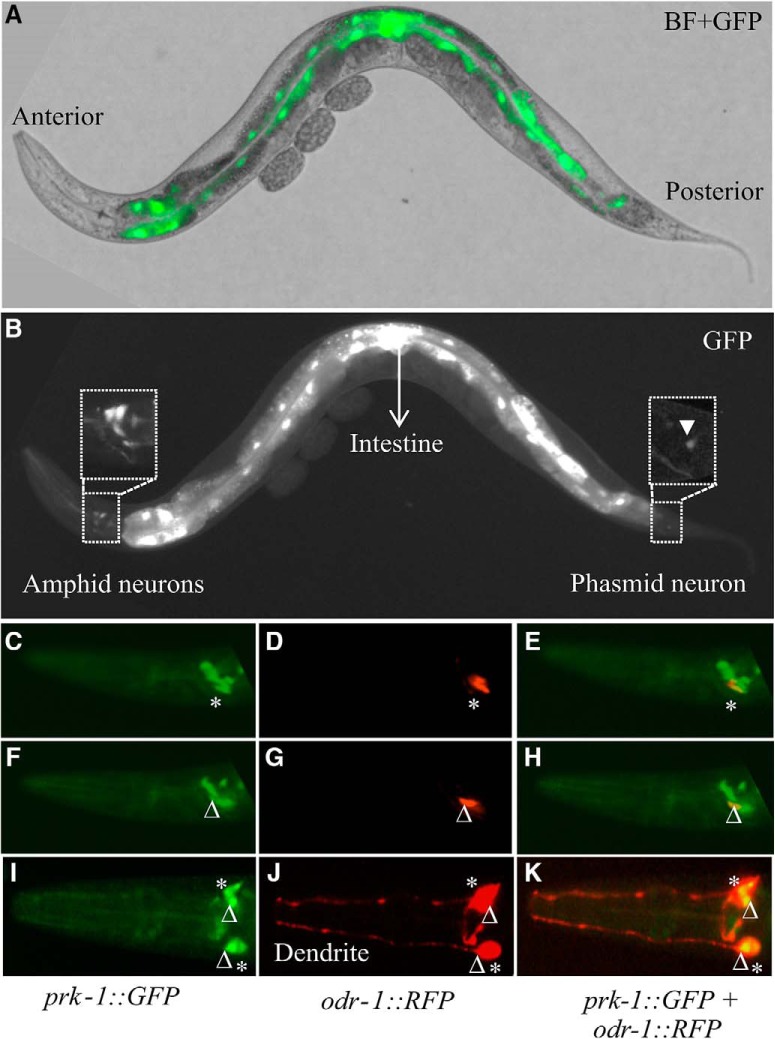
Expression pattern of *prk-1*. ***A***, ***B***, Lateral bright-field (BF) and fluorescent views of a representative adult animal from the transgenic *prk-1::GFP* strain expressing GFP in amphid and phasmid neurons and in the intestine. Areas indicated by rectangles have been magnified to show GFP expression in the amphid and phasmid neurons (white arrowhead). ***C***–***H***, Lateral views from different focal planes of a double transgenic animal expressing the *prk-1* promoter-driven GFP (***C***, ***F***) and the *odr-1* promoter-driven RFP marker (***D***, ***G***) in AWC (*) and AWB (Δ) neurons, as well as their colocalization in the merged picture (***E***, ***H***). ***I–K***, Dorsal view from another double transgenic animal to demonstrate that the *prk-1* and *odr-1* promoter-driven markers are expressed on both sides of the head.

To confirm that PRK kinases are present in those amphid neurons, whose activities are suppressed by the PIM inhibitors, we wanted to analyze the PRK expression patterns in more detail. Unfortunately there are no antibodies available against PRKs to allow direct detection of these proteins. However, using the *prk-1::GFP* reporter strain, we were able to confirm previous data on *prk-1* promoter-driven GFP expression in the intestine as well as in the head and tail neurons (Fig. 8*A*,*B*). A higher magnification revealed that the reporter expression is detected in a few amphid neurons and in one phasmid neuron. To obtain further evidence for the presence of PRK-1 expression in amphid neurons, we crossed the *prk-1::GFP* strain with the *odr-1::RFP* marker strain, where fluorescence is specifically detected in AWB and AWC neurons. Imaging of animals coexpressing these reporters revealed colocalized expression patterns for *prk-1::GFP* and *odr-1::RFP* in these two types of neurons on both sides of the heads (Fig. 8*C*–*K*). These results support our observations from the olfactory assays, suggesting that PRK-1 regulates olfactory responses to volatile attractants and repellents via AWB and AWC neurons.

## Discussion

In this study, we have shown that the *C. elegans* PRKs are true orthologs for the mammalian PIM family kinases, as demonstrated by comparison of the properties of PIM-1, PRK-1 and PRK-2. Based on our *in vitro* kinase assay data, PRKs are able to target also such known PIM substrates as NFATC1, which are not expressed in *C. elegans*, confirming the functional conservation of the enzymatic activity. The amino acid sequences within the kinase domains of PIMs and PRKs are highly conserved, especially at the ATP-binding site and the catalytically active site. However, the sequences of different lengths outside the kinase domains do not show any homology between the mammalian and *C. elegans* proteins, and are not conserved between PRK-1 and PRK-2, either. Thus, these sequences may allow more individual interactions of distinct PRK family members with other proteins.

The catalytic activity of the mammalian PIM kinases can be blocked by selective ATP-competitive inhibitors, such as DHPCC-9. Crystallization studies have revealed that within the ATP-binding pocket of murine PIM-1, DHPCC-9 forms hydrophobic contacts to the highly polar region including Lys67 (Akué-Gédu et al., 2009), which in *C. elegans* PRK-1 corresponds to Lys87 and in PRK-2 to Lys60. Our data indicate that the structures of the ATP-binding pockets of PIM orthologs are so well conserved from mammals to *C. elegans* that it is possible to use PIM-selective inhibitors as tools to study physiological functions of both PRK-1 and PRK-2. However, in the absence of structural data on the interactions of PIM inhibitors with PRKs, we cannot completely rule out any off-target effects, which is why we found it important to use three structurally unrelated PIM-inhibitory compounds in our assays, including also SGI-1776 and AZD-1208.

Using the pan-PIM inhibitors, we have characterized the roles of *C. elegans* PRKs in regulation of chemosensation. According to our data from chemotaxis assays, all the three tested PIM-selective inhibitors have very similar effects on *C. elegans* chemosensation. All of them can in a dose-dependent fashion block chemotaxis toward butanone sensed by AWC^ON^ neurons, with complete suppression seen already at sublethal concentrations of the inhibitors. In addition, some suppressive effects are observed toward benzaldehyde sensed by both AWC^ON^ and AWC^OFF^ neurons. By contrast, responses to 2,3-pentanedione sensed by AWC^OFF^ neurons or pyrazine and diacetyl sensed by AWA neurons remain unaffected, whereas some suppression is detected in chemotaxis to trimethylthiazole sensed by both AWA and AWC neurons. These results strongly suggest that PRK activity is essential for the olfactory functions of AWC^ON^ neurons, but is dispensable for sensations via AWA or AWC^OFF^ neurons. These conclusions are supported by our observations, according to which the *prk-1(pk86)* mutant animals lacking PRK-1 activity are unable to sense butanone, although they respond normally to isoamyl alcohol. The suppressive effects of PIM inhibitors on butanone sensing are rapid and reversible, suggesting that PIM kinases directly modulate activities of the neurons involved instead of, e.g., regulating their development. In addition, the effects are dependent on the dosage of the attractant, as high butanone concentrations can overcome the suppression, whereas lower concentrations reduce the chemotaxis in both control- and drug-exposed animals.

As demonstrated in the presence of the repellent 1-octanol, PIM inhibitors or lack of PRK-1 activity can also block aversive responses to volatile compounds via AWB neurons. By contrast, responses to water-soluble gustatory attractants, such as NaCl and KCl via ASE neurons, are not affected. Combinatory assays with both olfactory and gustatory cues revealed that chemotaxis toward attractive odorants is efficiently blocked by a barrier of water-soluble metal compounds such as copper sensed via ASH neurons. Although PIM inhibitors do not interfere with such aversive gustatory responses, they selectively reduce AWC^ON^ -dependent olfactory sensing of butanone also in this setting.

In conclusion, our data indicate that PRK activity is essential for olfactory attraction via AWC^ON^ neurons and repulsion via AWB neurons, but dispensable for gustatory sensations via ASE and ASH neurons. Interestingly, because the *prk-2(ok3069)* mutants lacking PRK-2 activity did not have similar problems in olfactory sensing as *prk-1(pk86)* mutants, this suggests that PRK-1 is the family member that is mainly responsible for the observed effects of pan-PIM inhibitors on olfaction. This conclusion is further supported by our observations on the *prk-1* promoter-driven reporter expression in both AWB and AWC neurons. Here it should be noted that AWC^ON^ and AWC^OFF^ neurons are structurally similar, but differ from each other by randomly selected expression of the G-protein-coupled seven transmembrane receptor (STR-2) on either right or left side of the animals, resulting in functional differences in the ability of these neurons to detect olfactory cues (Pierce-Shimomura et al., 2001; Wes and Bargmann, 2001). As PRK-1 is expressed in AWC neurons on both sides of the animals and as PIM inhibitors disrupt sensory functions of only AWC^ON^ neurons expressing STR-2, the putative connection between PRK-1 and STR-2 activities should be analyzed in more detail.

The observed effects on olfaction, but not gustation are well in line with our previous observations that during mouse embryogenesis, PIM kinases are expressed in the olfactory epithelium, but not in any gustatory organs (Eichmann et al., 2000). Although the sensory structures do not show any similarity between mice and nematodes, the odorant-induced intracellular signaling pathways of olfactory neurons are surprisingly similar between them (Pellegrino and Nakagawa, 2009). Thus, in the future it would be interesting to test whether mice lacking *pim* genes or treated with PIM inhibitors have problems in olfactory sensing. In case PIM inhibitors were able to reduce also human olfactory responses, this phenomenon could even be used as a biomarker for the efficacy of PIM-targeted therapies that are currently being developed against several types of hematological or solid cancers.

In developing mouse embryos, PIM kinases are expressed also in the neural retina (Eichmann et al., 2000), suggesting that PIM inhibition may negatively affect not only olfaction, but also vision. Indeed, evidence for that has already been obtained from studies with *D. rerio* zebrafish embryos, where low and high PIM expression levels were observed in the neural retina at Day 3 and 5 postfertilization, respectively (Yin et al., 2012). Furthermore, genetic or pharmacological inhibition of PIM expression or activity significantly diminished the visual functions of the embryos without affecting retinal morphology. Thus, these data together with our observations suggest that PRKs selectively affect sensory functions in an evolutionarily conserved fashion, with implications also in mammalian species. However, further studies are still needed to identify the relevant PIM substrates involved and to dissect the molecular mechanisms in more detail.

## Synthesis

Reviewing Editor: Cheryl Sisk, Michigan State University Decisions are customarily a result of the Reviewing Editor and the peer reviewers coming together and discussing their recommendations until a consensus is reached. When revisions are invited, a fact-based synthesis statement explaining their decision and outlining what is needed to prepare a revision will be listed below. The following reviewer(s) agreed to reveal their identity: Steven Munger. Note: If this manuscript was transferred from JNeurosci and a decision was made to accept the manuscript without peer review, a brief statement to this effect will instead be what is listed below.

This is significantly improved manuscript. It now includes important controls that were lacking in the first version. Furthermore, the conclusions and interpretations are conservative and supported by the data. A few additional revisions are suggested; these will require only editorial review.

1. Bar graphs in Figures 6 and 7 should be changed to the more informative box and whisker plots (as are now used in Figure 5).

2. For statistical analysis, the relevant post hoc test used should be indicated in the figure legends.

3. Please include in the methods section more details in the description of the mutant prk-1 and prk-2 alleles. Where are the predicted deletions and Tc1 insertion in the prk genes? Are they predicted to affect the ORF, or are they in introns or in the promoter?
